# Attitudes towards faecal immunochemical testing in patients at increased risk of colorectal cancer: an online survey of GPs in England

**DOI:** 10.3399/bjgp18X699413

**Published:** 2018-10-09

**Authors:** Christian von Wagner, Sandro Stoffel, Madeleine Freeman, Helga Laszlo, Brian D Nicholson, Jessican Sheringham, Dorothy Szinay, Yasemin Hirst

**Affiliations:** Research Department of Behavioural Science and Health;; Research Department of Behavioural Science and Health;; Research Department of Behavioural Science and Health;; UCLH Cancer Collaborative, London.; Nuffield Department of Primary Care Health Sciences, University of Oxford, Oxford.; Department of Applied Health Research, University College London, London.; Research Department of Behavioural Science and Health;; Research Department of Behavioural Science and Health;

**Keywords:** colorectal cancer, cross-sectional survey, faecal immunochemical test, 2-week wait, primary care, symptomatic

## Abstract

**Background:**

There is increasing interest in using a quantitative faecal immunochemical test (FIT) to rule out colorectal cancer (CRC) in patients with high-risk symptoms in primary care.

**Aim:**

This study aimed to investigate GPs’ attitudes and willingness to use a FIT over an urgent 2-week wait (2WW) referral.

**Design and setting:**

A cross-sectional online survey involving 1024 GPs working across England.

**Method:**

Logistic regression models were used to explore the likelihood of GPs using a FIT instead of a 2WW referral, and reported using odds ratios (ORs) and 95% confidence intervals (95% CIs).

**Results:**

Just over one-third of GPs (*n* = 365) preferred to use a FIT as a rule-out test over a 2WW referral. GPs were more willing if they were: aged 36–45 years (OR 1.59 [95% CI = 1.04 to 2.44]); 46–55 years (OR 1.99 [95% CI = 1.14 to 3.47]); thought a FIT was highly accurate (OR 1.63 [95% CI = 1.16 to 2.29]); thought patients would benefit compared with having a colonoscopy (OR 2.02 [95% CI = 1.46 to 2.79]); and were highly confident about discussing the benefits of a FIT (OR 2.14 [95% CI = 1.46 to 3.16]). GPs were less willing if they had had >10 urgent referrals in the past year (OR 0.62 [95% CI = 0.40 to 0.94]) and thought that longer consultations would be needed (OR 0.61 [95% CI = 0.44 to 0.83]).

**Conclusion:**

The study findings suggest that the acceptability of using a FIT as a rule-out test in primary care is currently low, with less than half of GPs who perceived the test to be accurate preferring it over colonoscopy. Any potential guideline changes recommending a FIT in patients with high-risk symptoms, instead of urgent referral to rule out CRC, are likely to require intensive supporting educational outreach to increase GP confidence in the accuracy and application of a FIT in this context.

## INTRODUCTION

Colorectal cancer (CRC) is the third most common cancer in the UK and the second biggest cancer killer.^1^ There are now organised cancer screening programmes in most countries across Europe and North America, including the UK,^2^ although the vast majority of CRCs are still diagnosed in response to symptoms.^3^^,^^4^ Even high-risk symptoms — outlined in Box 1, which summarises guidance for GPs who suspect patients of having CRC — only have a positive predictive value of 3–4%, meaning that the vast majority of patients who undergo an investigation will not have cancer.^5^^,^^6^

Box 1. **Guidance summary for suspected colorectal cancer**

1. [NG12^5^] Refer adults using a suspected cancer pathway referral (for an appointment within 2 weeks) for colorectal cancer if:
  - they are aged ≥40 years with unexplained weight loss and abdominal pain; or
  - they are aged ≥50 years with unexplained rectal bleeding; or
  - they are aged ≥60 years with:
    - iron-deficiency anaemia; or
    - changes in their bowel habit; or
  - tests show occult blood in their faeces.
2. [NG12^5^] Consider a suspected cancer referral pathway referral (for an appointment within 2 weeks) for colorectal cancer in adults with a rectal or abdominal mass.
3. [NG12^5^] Consider a suspected cancer referral pathway referral (for an appointment within 2 weeks) for colorectal cancer in adults aged <50 with rectal bleeding and any of the following unexplained symptoms or findings:
  - abdominal pain;
  - change in bowel habit;
  - weight loss;
  - iron-deficiency anaemia.
4. [DG30^15^] The OC-Sensor, HM-JACKarc, and FOB Fold quantitative faecal immunochemical tests are recommended for adoption in primary care to guide referral for suspected colorectal cancer in people without rectal bleeding who have unexplained symptoms but do not meet the criteria for a suspected cancer pathway referral outlined in section 1, 2, and 3 [July 2017].
  - Results should be reported using a threshold of 10 μg of haemoglobin per gram of faeces. Companies should provide advice about the performance characteristics of the assays to laboratories, and ensure standardisation of results.

For patients who can tolerate the procedure, colonoscopy is the current gold-standard test to rule out and detect CRC; it is accessed via the fast-tracked 2-week wait (2WW) pathway; that is, the maximum wait-time target in the UK for patients with suspected cancer to be first seen by a specialist in secondary care.^7^^,^^8^ Notwithstanding the potential human cost of an invasive procedure, colonoscopy could miss up to 10% of cancers and carries a small risk of complications.^9^ In addition, currently, colonoscopy resources in the UK are stretched to their limits.^10^^–^12 The Achieving World-Class Cancer Outcomes cancer strategy 2015–2020 advocates increased access for GPs to diagnostic point-of-care tests in primary care;^13^ however, only about half of patients with CRC receive a test in primary care before an urgent referral for suspected CRC.^14^

In July 2017, the National Institute for Health and Care Excellence (NICE) updated the *Suspected Cancer: Recognition and Referral* guideline (NG12) by publishing a new diagnostic guidance (DG30).^15^ DG30 recommended that a quantitative faecal immunochemical test (FIT) be used as a primary care triage test for patients with ‘low risk but no risk’; that is, patients who do not meet the 2WW referral criteria.^5^ There has also been interest in using a FIT as a rule-out test for patients with high-risk symptoms of CRC who do qualify for 2WW referral, based on its negative predictive value range of 99.8–100% with a threshold of 10 μg faecal haemoglobin (FHb) with a single sample.^15^^,^^16^ The potential recommendation is being investigated in ongoing studies.^17^^,^^18^ So far, clinical studies suggest that low or undetectable FHb may rule out significant bowel disease,^19^^,^^20^ and a recent systematic review concluded that approximately 75% of patients who are symptomatic could avoid colonoscopy if triaged beforehand with a FIT.^21^

If a FIT is implemented in primary care as a rule-out test for patients who have high-risk CRC symptoms, it could reduce the need for a 2WW referral for patients who receive a negative test result, and reduce the pressure on colonoscopy services.^16^^,^^19^^,^^22^^,^^23^ However, little is known about how GPs would use a FIT as a rule-out test of CRC and the acceptability of the test kit. Successful adoption of the FIT in primary care, therefore, will rely on GPs’ attitudes about the test and their role in implementing its use. The aim of this online survey was to identify the attitudes and beliefs of UK GPs regarding use of a FIT as a rule-out test in order to inform clinical decision makers during implementation of the FIT in primary care. The survey included questions on:

- existing awareness and use of a FIT as a rule-in test;
- attitudes and beliefs towards the FIT;
- GP demographics; and
- practice characteristics.

**How this fits in**

There is an increase in demand for colonoscopies, which could have a negative impact on the urgent referral timeline — 2-week wait (2WW) — applied in England. A faecal immunochemical test (FIT) with a low or undetectable faecal haemoglobin could be used as a triage test in primary care to rule out colorectal cancer and significantly reduce the number of unnecessary colonoscopies among patients who are symptomatic. The findings presented here indicate that only just over one-third of GPs across England would prefer to use a FIT over a referral to the 2WW pathway with a diagnostic colonoscopy to rule out suspected lower gastrointestinal cancers among patients with symptoms; even among GPs who perceived FITs to be accurate, more than half would still prefer a 2WW over a FIT. Initiatives to implement the FIT in primary care as a rule-out test need to be accompanied by efforts to improve GPs’ perception of test accuracy, as well as addressing concerns about increased consultation time and other implementation or organisational issues.

## METHOD

### Study design and sample

In December 2017, 14 100 GPs from England were invited to take part in a 10-minute online survey using M3 Global Research’s panel of GP workforce via https://www.doctors.net.uk/in the UK (*n* = 41 935). M3 Global Research is a research company with a focus on health professionals and has the largest global International Organization for Standardization-certified physician community in the world. GPs were eligible if they were working in England at the time the study took place. M3 Global Research randomly invited GPs to the study. GPs were offered an industry-standard honorarium (£15 per GP) based on the length of the survey.

Consenting GPs were provided with a hypothetical vignette for the potential use of a FIT in primary care as a point-of-care test to rule out CRC in patients with symptoms that would normally qualify them to be referred through the 2WW pathway. Responders were told that FIT as a rule-out test would miss a very low number of CRCs, comparable to that of a colonoscopy, and *‘... having a very high negative predictive value and minimal false negatives’*. No detailed statistical information on test specificity or sensitivity was provided; this was done to avoid confusion relating to different uses of the FIT and the lack of recommendations for patients with high-risk symptoms. Instead, the focus was on the comparison with a colonoscopy because, if implemented, a FIT will be used as an alternative to the 2WW referral pathway. The vignette was evaluated by the research team for its consistency, accuracy, and relevancy. Detailed information is available from the authors on request.

### Measures

#### GP characteristics

GP characteristics were included based on previous research.^24^ GPs provided:

- age (categorised in bands);
- sex;
- years of experience as an active GP;
- role in their practice;
- engagement in implementation/budget planning;
- role as a cancer lead;
- engagement in research; and
- practice size (number of registered patients and GPs working at the practice).

They also indicated the number of 2WW referrals they had made over the previous 12 months.

#### Using a FIT in primary care

GPs were asked to choose their preferred pathway for patients with high-risk CRC symptoms, from the following options:

- ask the patient to complete a FIT to rule out CRC before completing the 2WW referral; or
- implement the 2WW referral for suspected CRC (no FIT).

They were also asked *‘How accurate do you think the FIT is as point-of-care test to rule out CRC compared to a colonoscopy?’* Each item was measured on a 4-point scale: very accurate, quite accurate, not very accurate, not at all accurate; unsure was also included as an additional response option.

GPs’ perceived confidence around discussing the benefits and harms of FIT with patients was measured. Two items assessed GPs confidence of discussing negative and positive FIT results respectively. Each item was measured on a 4-point scale: very accurate, quite accurate, not very accurate, not at all accurate; unsure was also included as an additional response option.

GPs were asked to consider to what extent they believed patients would benefit from completing a FIT compared with having a colonoscopy, and whether they would benefit from using a FIT as a rule-in or rule-out test. They were also asked about their beliefs about the practicalities of the FIT, and about their role in implementing a FIT in primary care; this last was measured using 5 items. Each item was measured with a 4-point scale (‘strongly agree’, ‘agree’, ‘disagree’, and ‘strongly disagree’) and included ‘don’t know’.

Responders were asked whether they were aware of the uses of FIT as a rule-in test for patients at low risk of having CRC, and its use as a rule-out test among patients at high risk of having CRC. Previous experience of using a faecal occult blood test (FOBT) or FIT in accordance with the NICE guidelines was measured.

GPs were presented with three categories that included several factors relating to the test, their organisational culture, and implementation of the FIT in primary care. They were asked to choose the most important category and to rate the top three most important factors that they, personally, think are important if FIT is implemented as a rule-out test.

### Statistical analysis

For ease of interpretation, responses were dichotomised, by combining ‘strongly agree’ and ‘agree’ versus ‘strongly disagree’, ‘disagree’, and unsure/don’t know. Descriptive statistics were used to demonstrate GP characteristics, along with a broad description of their attitudes and beliefs towards the implementation of a FIT. The frequency of the top-ranked items was used to order the importance of each factor relating to the implementation of a FIT in primary care. Only the top-ranked factor was explained in detail; information on the other factors is available from the authors on request.

Unadjusted logistic regression analyses were used to identify the potential factors associated with GPs’ preference to use a FIT as a rule-out test in primary care. The unadjusted odds ratios (ORs) and confidence intervals (95% CIs) are available from the authors on request. A final adjusted multivariable logistic regression model was used to test for all the factors that were identified in univariate regression analyses testing associated with the preference for using a FIT in primary care for patients who have high-risk CRC symptoms, as opposed to the 2WW referral without a FIT. The significance level for our analysis was *P*<0.05. All analyses were conducted using Stata (version 14.0).

## RESULTS

### Population characteristics

Of 14 100 invitees, 1351 (9.6%) GPs responded to the invitation during the data-collection period over 4 weeks. Of those, 209 started the survey but did not complete it and 118 were excluded because they did not qualify or had completed this survey on a previous occasion. In total, 1024 GPs from England successfully completed the survey; this equates to 7.3% of the total number of invitees. Table 1 gives an overview of GP characteristics.

**Table 1.** GP characteristics

**Characteristic**	**Study population (*n*= 1024), *n* (%)**	**Estimated GPs in England, 2017,^a^*n* (%)**
**Age in years**		
≤35	225 (22.00)	8374 (21.20)
36–45	453 (44.24)	12 827 (32.50)
46–55	217 (21.20)	10 673 (27.00)
≥56	129 (12.60)	7637 (19.30)

**Sex**		
Male	545 (53.22)	17 937 (45.20)
Female	479 (46.78)	21 755 (54.80)

**Employment status**		
Trainee	40 (3.91)	
Full time	283 (27.64)	
Part time	265 (25.88)	
Locum	163 (15.92)	
Partner	261 (25.49)	
Other	12 (1.17)	

**Geographical region**		
London	196 (19.14)	7592 (10.49)
Midlands and East of England	188 (18.36)	12161 (28.68)
South of England	265 (25.88)	10702 (25.25)
North of England	375 (36.62)	11934 (28.15)

**Experience in years**		
<10	477 (46.58)	
≥10	547 (53.42)	

**Has a role in budget setting**		
No	787 (76.86)	
Yes	237 (23.14)	

**Has a specialism**		
No	762 (74.41)	
Yes	262 (25.59)	

**Actively involved in research**		
No	891 (87.01)	
Yes	133 (12.99)	

**Patients registered at practice**		
<5000	173 (16.89)	
5000–10 000	390 (38.09)	
>10 000	461 (45.02)	

**GPs working in practice**		
<5	306 (29.88)	
5–10	557 (54.39)	
>10	161 (15.72)	

**CRC 2WW referrals in the previous 12 months**		
<5	153 (14.94)	
5–10	440 (42.97)	
>10	431 (42.09)	

**Aware of FIT as a rule-in test**		
No	592 (57.81)	
Yes	432 (42.19)	

**Aware of FIT as a rule-out test**		
No	788 (76.95)	
Yes	236 (23.05)	

**Have used FOBT in line with NG12^5^**		
No	466 (45.51)	
Yes	558 (54.49)	

**Have used FIT in line with DG30^15^**		
No	922 (90.04)	
Yes	102 (9.96)	

a *General and Personal Medical Services, England High Level March 2017, Provisional Experimental Statistics.^25^ 2WW* = *2-week wait. CRC* = *colorectal cancer. FIT* = *faecal immunochemical test. FOBT* = *faecal occult blood test.*

Of the 1024 responders, 42.2% (*n* = 432) were aware of the use of a FIT as a rule-in test for patients with low-risk symptoms, but only 23.0% (*n* = 236) were aware that a FIT may also be used as a rule-out test to exclude CRC. Approximately half of the GPs (54.4%) used an FOBT for patients with low-risk symptoms (*n* = 558) in line with national guidance; only 10.0% (*n* = 102) had used a FIT since the guidance update of August 2017.

### Use of FIT as a rule-out test

In total, 35.6% of responders (*n* = 365) reported that they would prefer to use a FIT as a rule-out test compared with 64.4% (*n* = 659), who stated that they would prefer the 2WW referral without a FIT. Table 2 outlines the fully adjusted analyses, which showed that responders were more likely to prefer FIT if they:

- were aged 36–45 years (OR 1.59, 95% CI = 1.04 to 2.44);
- were aged 46–55 years (OR 1.99, 95% CI = 1.14 to 3.47);
- agreed that a FIT is highly accurate to rule out CRC (OR 1.63, 95% CI = 1.16 to 2.29);
- were confident discussing the benefits of a FIT (OR 2.15, 95% CI = 1.46 to 3.16); and
- believed that patients would benefit from completing a FIT as opposed to a colonoscopy (OR 2.02, 95% CI = 1.46 to 2.79).

**Table 2.** Factors associated with GPs’ preference to use FIT over 2WW

	**Test**		**Adjusted OR**	**95% CI**

	**2WW, *n* (%)**	**FIT, *n* (%)**		
Overall	659 (64.0)	365 (35.6)		

**GP characteristics**				
Age, years				
≤35	164 (72.9)	61 (27.1)	Ref.	
36–45	288 (63.6)	165 (36.4)	1.59	1.04 to 2.44^a^
46–55	126 (58.1)	91 (41.9)	1.99	1.14 to 3.47^a^
≥56	81 (62.8)	48 (37.2)	1.47	0.78 to 2.78
Years active as a GP				
<10	326 (68.3)	151 (31.7)	Ref.	
≥10	333 (60.9)	214 (39.1)	1.00	0.68 to 1.47

CRC 2WW referrals in the last year				
<5	88 (57.5)	65 (42.5)	Ref.	
5–10	266 (60.5)	174 (39.5)	0.90	0.59 to 1.36
>10	305 (70.8)	126 (29.2)	0.62	0.40 to 0.94^a^

**FIT awareness and previous experience**				
Previous experience using FIT for low-risk patients				
No	316 (67.8)	150 (32.2)	Ref.	
Yes	343 (61.5)	215 (38.5)	1.08	0.81 to 1.45
Perceived test accuracy				
Not at all, not very, unsure	284 (78.9)	76 (21.1)	Ref.	
Quite, very	375 (56.5)	289 (43.5)	1.63	1.16 to 2.29^b^

**Confidence discussing FIT with high-risk patients**				
Confidence discussing benefits				
Not at all, not very, unsure	438 (78.5)	120 (21.5)	Ref.	
Quite, very	221 (47.4)	245 (52.6)	2.15	1.46 to 3.16^b^
Confidence discussing harms				
Not at all, not very, unsure	439 (73.0)	162 (27.0)	Ref.	
Quite, very	220 (52.0)	203 (48.0)	0.98	0.68 to 1.41
Confidence discussing negative results				
Not at all, not very, unsure	360 (75.5)	117(24.5)	Ref.	
Quite, very	299 (54.7)	248 (45.3)	1.09	0.76 to 1.55
Confidence discussing positive results				
Not at all, not	218 (75.5)	57 (20.7)	Ref.	
Quite, very	441 (58.9)	308 (41.1)	1.08	0.72 to 1.62

**Beliefs about patients’ experience and thoughts about FIT**				
Patients would benefit from completing FIT compared with colonoscopy				
Strongly disagree, disagree	365 (79.2)	96(20.8)	Ref.	
Agree, strongly agree	294 (52.2)	269 (47.8)	2.02	1.46 to 2.79^a^
Patients will prefer FIT as a rule in test of CRC				
Strongly disagree, disagree	257 (72.8)	96 (27.2)	Ref.	
Agree, strongly agree	402 (59.9)	269 (40.1)	1.05	0.75 to 1.47
Patients will prefer FIT as a rule-out test				
Strongly disagree, disagree	215 (78.5)	59 (21.5)	Ref.	
Agree, strongly agree	444 (59.2)	306 (40.8)	1.25	0.85 to 1.84
Patients will perceive the test to be easy to complete at home				
Strongly disagree, disagree	88 (73.3)	32 (26.7)		
Agree, strongly agree	571 (63.2)	333 (36.8)	0.93	0.57 to 1.53

**Beliefs about GPs’ role in FIT implementation**				
I believe that implementation of FIT in Primary care is a legitimate part of my role				
Strongly disagree, disagree	244 (79.7)	62 (20.3)	Ref.	
Agree, strongly agree	415 (57.8)	303 (42.2)	1.13	0.74 to 1.73
I can easily integrate FIT into my existing work with patients who present with high-risk symptoms				
Strongly disagree, disagree	316 (76.3)	98 (23.7)	Ref.	
Agree, strongly agree	343 (56.2)	267 (43.8)	1.19	0.84 to 1.71
There is a strong need for FIT to be implemented in primary care as a point of care test for ruling out cancer				
Strongly disagree, disagree	287 (78.2)	80 (21.8)	Ref.	
Agree, strongly agree	372 (56.6)	285 (43.4)	1.17	0.79 to 1.71
I will need a longer consultation to be able to discuss why the patient will need to complete FIT				
Strongly disagree, disagree	201 (55.5)	161 (44.5)	Ref.	
Agree, strongly agree	458 (69.2)	204 (30.8)	0.61	0.44 to 0.83^a^

a P<*0.05.*

b P<*0.005. 2WW* = *2-week wait. CRC* = *colorectal cancer. FIT* = *faecal immunochemical test. OR* = *odds ratio.*

In contrast, beliefs about longer consultation time (OR 0.61, 95% CI = 0.44 to 0.83) and having >10 CRC 2WW referrals in the previous year (OR 0.62, 95% CI = 0.40 to 0.94) were negatively associated with preferring a FIT over the 2WW referral.

### Early adoption of FIT as a rule-out test in primary care

**Figure 1.**
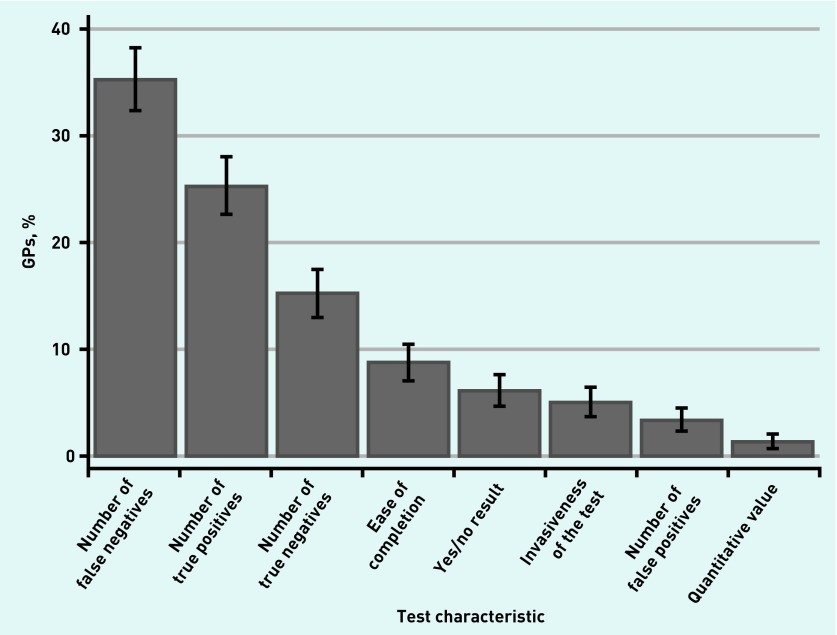
***Factors cited by GPs (n = 1024) in their decision to use a faecal immunochemical test in primary care to test for colorectal cancer in patients with high-risk symptoms.***

Of the responders, 60.5% (*n* = 620) stated that test characteristics would be the most important factor in their decision to use a FIT if it is implemented in primary care; organisational culture and implementation characteristics were only endorsed by 14.5% (*n* = 148) and 25.0% (*n* = 256) of the responders, respectively. Among test characteristics, 35.3% ranked the number of false negatives (*n* = 361) as the main factor that would encourage them to ask patients with high-risk symptoms to complete a FIT; this was followed by 25.3% (*n* = 259), who ranked the number of true positives, and 15.1% (*n* = 155) who cited the number of true negatives (Figure 1).

## DISCUSSION

### Summary

When consulting with patients with high-risk symptoms, only just over one-third of GPs across England would prefer to use a FIT to rule out CRC over referral to the 2WW pathway for a diagnostic colonoscopy; they highlighted the relative importance of test-specific information for the early adoption of a FIT in primary care. GPs’ likelihood of preferring a FIT increased if they believed the accuracy of the test to be high, were confident discussing the benefits with the patients, and strongly agreed that the patients would benefit from a FIT compared with having a colonoscopy. GP responders were less likely to prefer the FIT if they had had >10 2WW referrals for CRC in the previous 12 months and thought the consultations would be longer if FIT was implemented. The fact that the vast majority of GPs had not asked their patients to complete a FIT before means that their GP responder concerns about longer consultation times were not based in actual experience. These concerns present an anticipated barrier that should be considered by commissioners, who will provide pathways to FIT implementation in primary care. GPs who agreed that it is part of their role to support the implementation of a FIT, and that there is a strong need for a FIT, were no more likely to prefer the test than those who did not. Furthermore, there was no association between FIT preference and awareness of the use of FIT in primary care for patients classified as ‘low-risk but not no risk’.

### Strengths and limitations

As a relatively large, nationally representative sample, this survey study was able to highlight the barriers and facilitators of implementing the FIT in primary care for patients who may present with high-risk CRC symptoms. However, there were some notable limitations. Despite the large number of responders, the actual survey response rate was <10%, thereby restricting the generalisability of the findings; however, comparable response rates have been observed in previous studies based on primary care using similar methodologies.^24^^,^^26^

It is also worth noting that GPs only expressed their preference in the absence of clinical recommendations. The awareness of different uses of a FIT in symptomatic context prior to the survey was low; as such, the study was designed with caution to avoid confusion that might arise from the different thresholds and recommendations that exist for the different uses of a FIT.^15^^,^^18^^,^^21^

Most GPs in this survey prioritised test characteristics — and, specifically, test accuracy — when considering whether to use a FIT in patients with high-risk symptoms. However, it must be noted that, even among GPs who perceived the FIT to be accurate, only 35.6% would prefer to use it over a 2WW referral. This highlights that, even if current research projects showed that a FIT could rule out cancer with 100% accuracy,^17^^,^^18^ more work would need to be done to overcome other anticipated barriers.

Perceptions of FIT might be further confounded by a tendency to overemphasise test sensitivity (as revealed by the relative importance that GPs attributed to true positives), despite the fact that, in the context of a rule-out test, specificity (that is, true negatives and false positives) is equally important, particularly with future sustainability of endoscopy services in mind. One potential way to understand whether accuracy is the key factor in GPs’ decisions to use FIT over 2WW would be using discrete-choice studies, which will investigate test preferences based on FIT statistics (threshold, sensitivity, and specificity) and other test attributes, such as changes in consultation times.

At present, there are very little data on the proportion of test kits that are successfully completed and returned in the diagnostic context. Thus it is important to use insights from studies into barriers to completing similar tests for CRC screening to inform future research into FIT implementation as a diagnostic test. Specifically, it has been shown that non-white ethnic groups, people with low health literacy, non-English speakers, and those who do not engage with the information are less likely to complete the test kit.^27^^–^30 As the FIT, if implemented as a rule-out test, would be aimed at patients with high-risk symptoms, it is imperative to address GPs’ concerns about their patients’ ability to complete the test kits and have appropriate safety-netting activities (for example, text-message reminders and follow-up appointments) in place in both primary and secondary care.

### Implications for research and practice

As the FIT will be implemented in CRC screening in England,^31^ there will be potentially three different clinical contexts (screening population, and patients with low- and high-risk symptoms) in which immunochemical testing is used. Each context could vary on a number of aspects; most notably, clinical thresholds used to determine follow-up referrals. Given that GPs’ awareness of using a FIT in both rule-in and rule-out contexts was low among the survey responders, in future, particular attention should be paid to raising GPs’ awareness of different thresholds used in different contexts, as well as patients’ experience of using a FIT and their understanding of the purpose of a FIT in all clinical contexts.

This national survey suggests that a majority of GPs would prefer to use the current default test. Concerns about additional pressures on consultation time should be addressed in anticipation of any changes to NICE’s guidelines. Qualitative interviews with GPs might gain additional insights about potential barriers and identify the most effective ways of supporting implementation of a FIT as a rule-out test for CRC. The current implementation of NICE guidelines for patients with low-risk symptoms will offer further observational evidence.
